# Supplementary material for: Vibrio cholerae Motility in Aquatic and Mucus-Mimicking Environments
Source: Appl Environ Microbiol. 2021 Sep 28;87(20):e01293-21. doi: 10.1128/AEM.01293-21 (PMC8478464; doi:10.1128/AEM.01293-21)
Supplement: Supplemental file 1 — Fig. S1 to S5, Table S1, Notes S1 and S2. Download AEM.01293-21-s0001.pdf, PDF file, 1.2 MB [file aem.01293-21-s0001.pdf]

Supplementary Information

Vibrio cholerae motility in aquatic and mucus-mimicking environments

Marianne Grognot, Anisha Mittal, Mattia Mah'moud, Katja M. Taute#
Rowland Institute at Harvard, 100 Edwin H. Land Blvd, Cambridge, MA 02142, USA
#corresponding author: taute@rowland.harvard.edu

Supplementary Figure 1

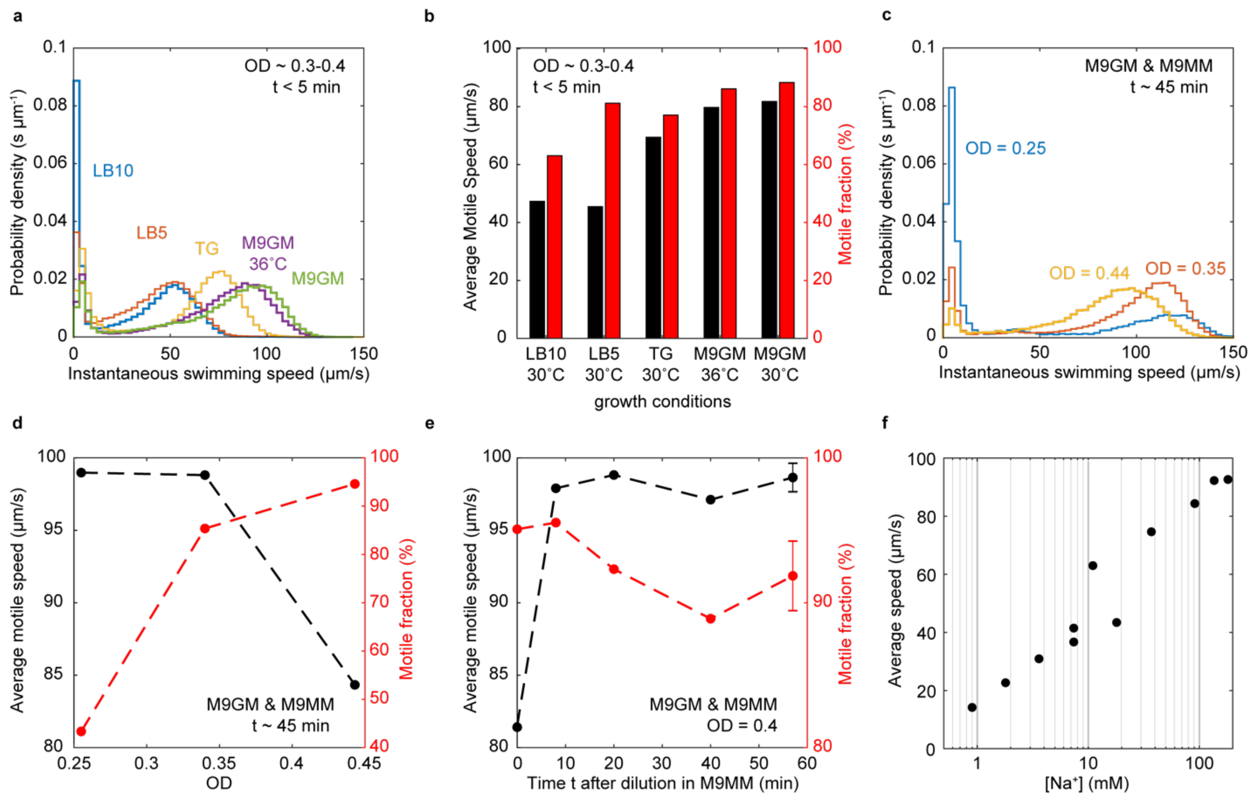

Supplementary Figure 1: Protocol optimization and characterization for *V. cholerae* 0395-NT motility assays.

a) Distribution of instantaneous swimming speeds for cultures grown to an OD of 0.30-0.40 in the specified media, diluted in the same medium, and tracked within 5 min.

b) Average speed and fraction of motile bacteria, defined by an average speed above 20 μm/s, for the same conditions as in panel a.

c) Distributions of instantaneous swimming speed for cultures grown in M9GM to a given OD, diluted into motility medium M9MM, and tracked after approximately 45 minutes.

d) Average speed and fraction of motile bacteria, weighted by trajectory duration, as a function of OD for bacteria grown in MGMM, diluted into M9MM, and tracked after 45 min. Dashed lines in panels d, e serve to guide the eye.

e) Average speed and fraction of motile bacteria, weighted by trajectory duration, grown in M9GM to an OD of 0.4 as a function of incubation time in M9MM before tracking. Where present, error bars represent the SD for three technical replicates. Both parameters are stable within 20-60 min. An incubation time of 45 min was selected for further experiments.

f) Dependence of average swimming speed on Na^+ concentration in M9MM, for bacteria grown in M9GM to an OD of 0.3-0.4 and incubated in the relevant motility medium for 45 min.

Supplementary Figure 2

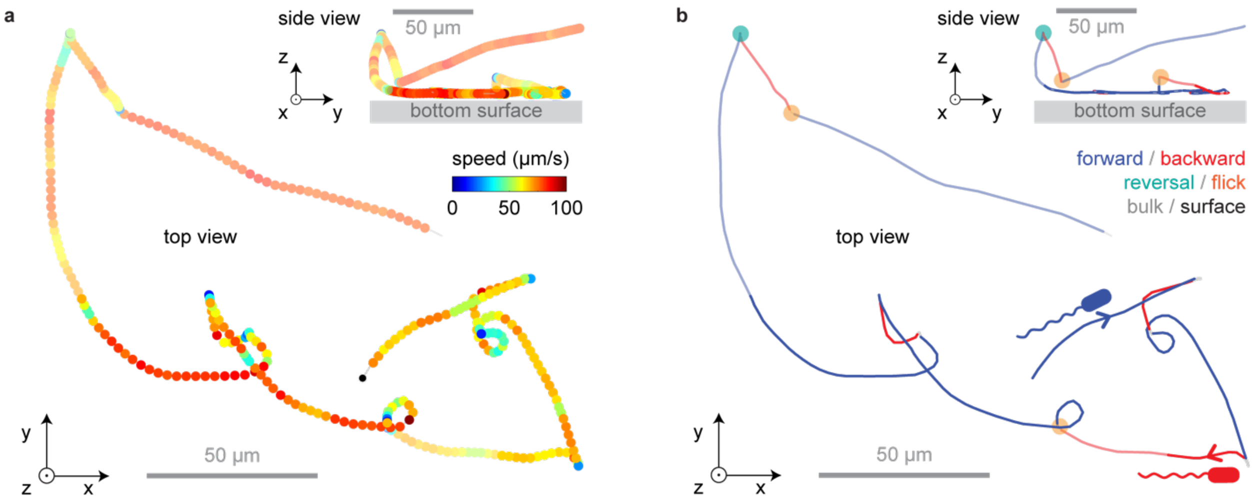

Supplementary Figure 2: Near-surface trajectory curvature confirms that *Vibrio cholerae* has a left-handed flagellum. Example trajectory color-coded by speed (a) and run orientation (b, red: backward, blue: forward), as seen from inside the chamber looking at the surface (top view), or from the side (side view). Bulk segments (further than 5 µm from the surface) are shown partially transparent. The run orientation is assigned based on the identity of turning events observed while away from the surface (orange: flick, teal: reversal). The top view shows right-handed trajectory curvature for forward and left-handed curvature for backward runs at the surface. This is consistent with forward runs being driven by a left-handed flagellum rotating CCW (as seen from behind the flagellum looking towards the cell body), so that the cell body rotates CW. The higher effective drag below the bacterium leads to the flagellum and the cell body effectively trying to roll sideways on the surface in opposite directions, producing a torque that causes a reorientation to the right(1).

Supplementary Figure 3

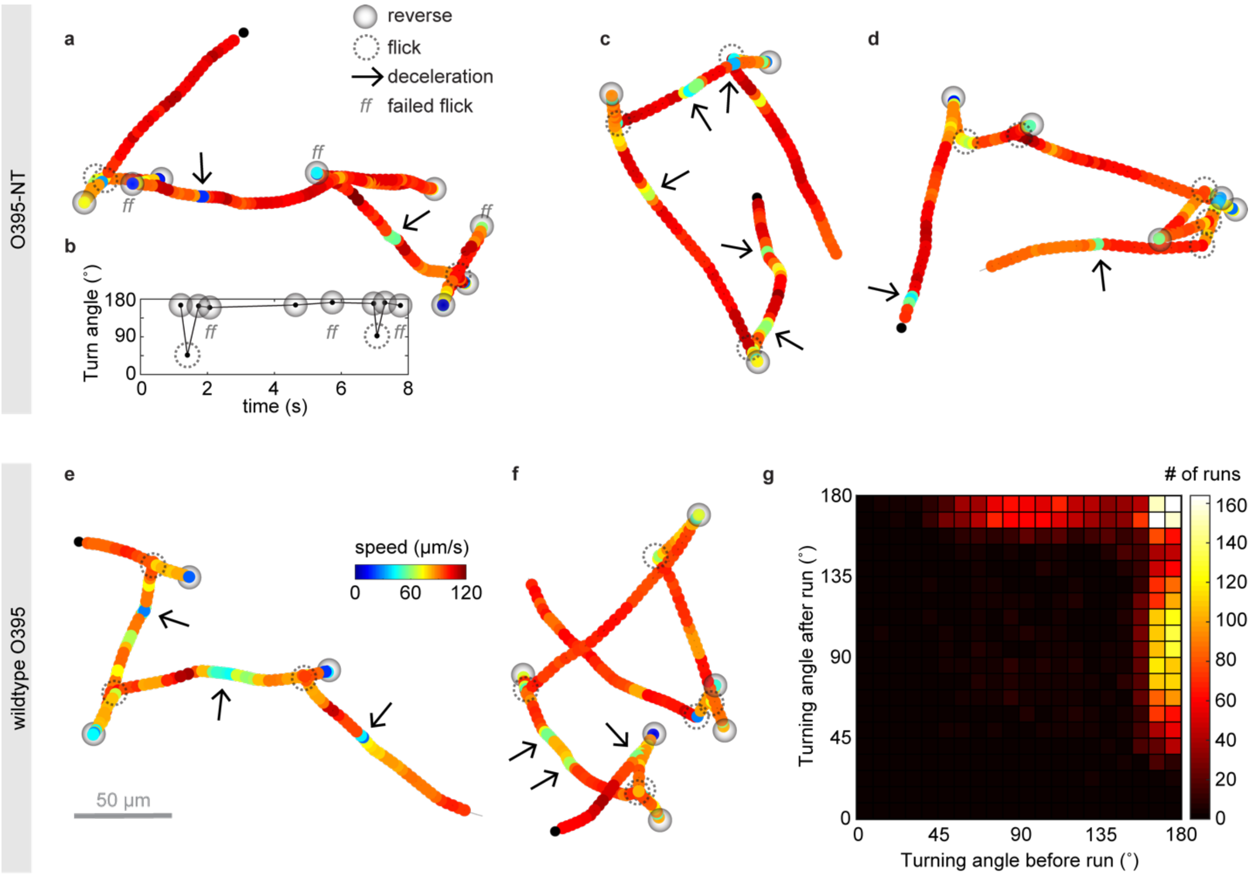

Supplementary Figure 3: Run-reverse-flick motility and deceleration events in O395-NT and wildtype O395. a,c,d) Further example trajectories of O395-NT showing run-reverse-flick motility and decelerations. b) Times series of turning angles for the trajectory in panel a show failed flick events. e,f) Example trajectories of wildtype O395 showing run-reverse-flick motility and decelerations. g) Bivariate histogram of consecutive turning angles for wildtype O395 also displays alternating flicks and reversals as well as consecutive reversals, consistent with run-reverse-flick motility.

Supplementary Figure 4

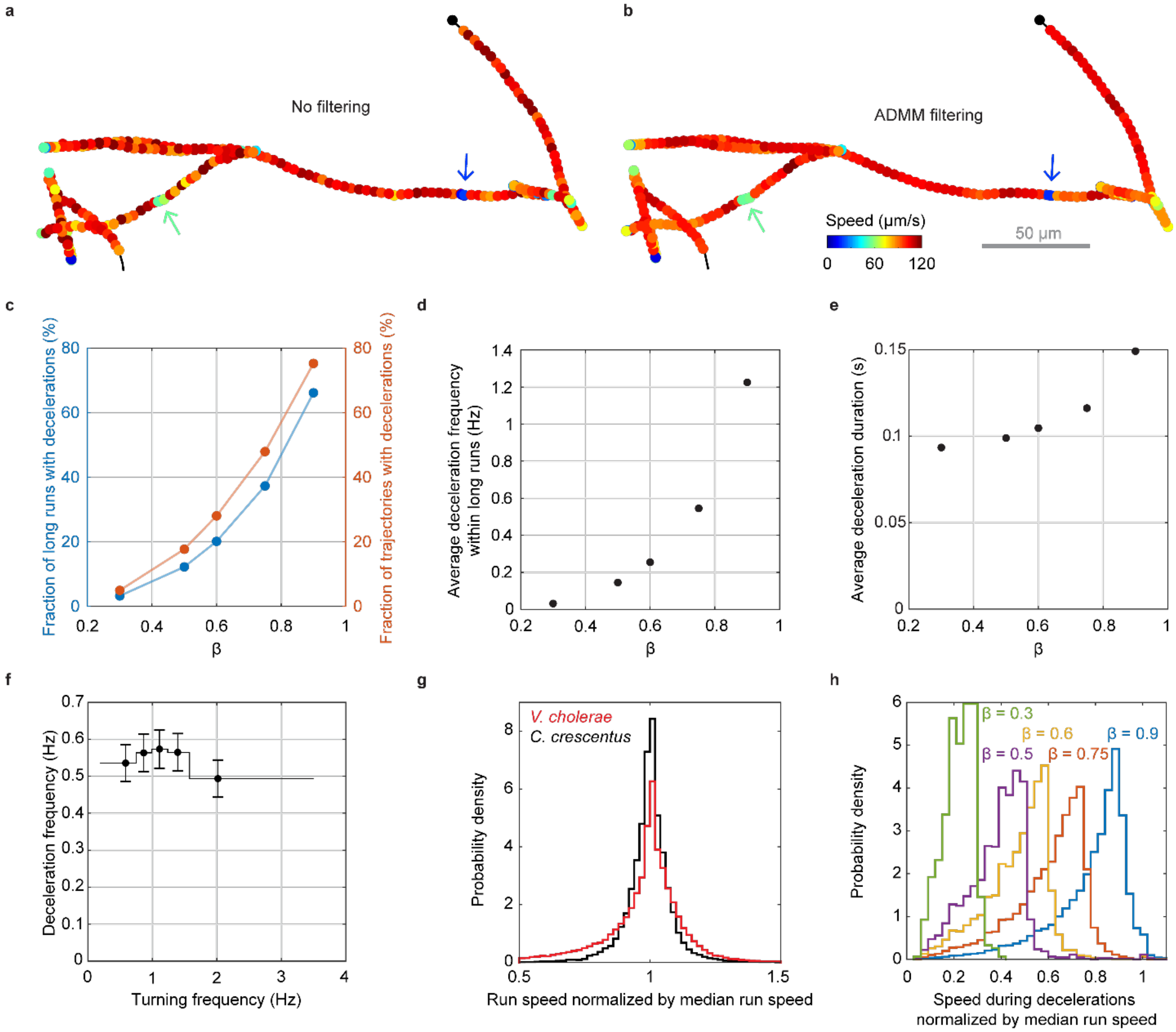

Supplementary Figure 4: Additional verification and characterization of deceleration events.

a) and b) An example trajectory for strain O395-NT shows pronounced deceleration events, marked by arrows, both before filtering (a) and after filtering (b).

c) Percentage of long runs (blue, with a duration of at least 10 frames) or bacterial trajectories with long runs (orange) that contain at least one deceleration event as a function of the threshold factor β. Dashed lines serve to guide the eye.

d) Deceleration frequency across all long runs as a function of the threshold factor β.

e) Average deceleration duration as a function of the threshold factor β.

f) Average deceleration frequency within the long runs of a trajectory, given the turning frequency of the trajectory. Error bars represent 95% confidence intervals. The Pearson correlation coefficient on the non-binned data is $r = -0.036 \pm 0.031$, with a p-value of 0.025.

g) Distribution of instantaneous run speeds normalized by each run's median speed for runs with a minimal duration of 0.33 s and a minimal average speed of 30 µm/s for *C. crescentus* and *V. cholerae*. Average run speeds are 53 µm/s for *C. crescentus* and 91 µm/s for *V. cholerae*.
h) Distribution of instantaneous swimming speeds during deceleration events, normalized by the run's median speed.

Supplementary Figure 5

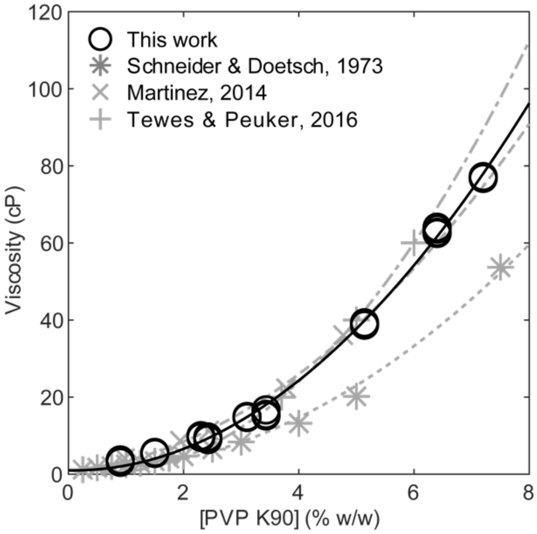

Calibration curve of macroscopic viscosity as a function of PVP K90 concentration for this study, and comparison to values from Schneider & Doetsch(2), Martinez et al.(3), and Tewes & Peuker(4). Lines represent fits of a second order polynomial constrained to match the viscosity of water at the appropriate temperature at a concentration of 0%.

Supplementary Table 1

Data-set	medium	μ (cP)	f (Hz)	replicates: bio. x tech. x dur. (min)	λ	v_{min} (μm/s)	N	T (s)	<v> (μm/s)	α	ν (Hz)
1	M9MM	0.98	30	3 x (3-5) x (2-2.2)	0.3	20	23,062	58,029	94.2	8	0.49
2	M9MM	0.98	30	3 x 1 x (2-2.2)	0.3	20	8,017	19,113	88.5	8	0.47
3	136 mM Na^+	0.98	30	1 x 1 x 2.5	0.3	20	2,695	6,991	92.3	8	0.45
	7.4 mM Na^+	0.98	15	1 x 1 x 5	0.8	10	5,089	11,996	36.7	8	0.60
	1.8 mM Na^+	0.98	15	1 x 1 x 5	0.8	10	4,083	14,467	22.7	8	0.74
4	TMN	0.98	30	1 x 3 x (1.7-2.5)	0.3	20					
	0.9% PVP/TMN	1.9	30	1 x 1 x (0.8-1.7)	0.3	15	1,407	4,539	48.7		
	1.5% PVP/TMN	3.9	30		0.3	12	608	2,512	33.1		
	2.2% PVP/TMN	8.1	30		0.3	10	543	1,919	26.1		
	4.5% PVP/TMN	31	15		0.3	6	488	2,714	14.3		
	6% PVP/TMN	54	15		0.3	6	383	5,195	10.8		
	6.7% PVP/TMN	68	15		0.3	5	557	8,054	9.4		
5	1.2% MUC5B/M9MM		30	1 x 2 x 1.7	0.3	5	2,327	6,027	57.4	8	0.49

Supplementary Table 1: Description and statistics of datasets. μ is the viscosity of the medium. μ is the macroscopic viscosity. f is the frame rate of video recordings. Replication information is presented as the number of biological replicates x the number of technical replicates x the recording duration. Technical replicates are recordings from different sample chambers. λ is the regularization parameter used for ADMM trajectory-smoothing (see Methods for details). v_{min} is the minimum trajectory-averaged swimming speed for being considered motile. N is the number of motile trajectories with a minimum duration of 1 s that are retained for turn detection analysis, except for dataset 4 where no turn detection was performed, and N is the number of motile trajectories. T is their total duration. <v> is the average swimming speed of the analyzed trajectories, and α is the threshold factor that was used for automated turn detection. ν is the average turning frequency, computed as the number of detected turn events divided by the total trajectory duration for the dataset. All datasets were obtained for strain O395-NT, except for dataset 2, which was obtained for O395 wildtype.

Supplementary Note 1

Dependence of the effective diffusion coefficient on relative run durations

Taktikos et al.(5) (Eq. 24) show that the effective diffusion coefficient D for a run-reverse-flick swimmer with forward and backward run durations τ_f and τ_b is given by

$$D = \frac{v^2}{3} \frac{\tau_b^2 - \tau_b\tau_f(1 - 2D_r\tau_b) + \tau_f^2(1 + 2D_r\tau_b)}{(\tau_b + \tau_f)(1 + 2D_r\tau_b)(1 + 2D_r\tau_f)}$$

where v is the swimming speed and D_r is the rotational diffusion coefficient characterizing the random change in orientation during runs. Our experiment yields measured values of τ_f^m = 0.62 s and τ_b^m = 0.174 s. In scenario 1, $\tau_f = \tau_f^m$ and $\tau_b = \tau_b^m$ as observed in our data. In scenario 2, $\tau_f = \tau_b$, such that $\tau_f = \tau_b = (\tau_f^m + \tau_b^m)/2$, i.e., the overall turning frequency is unchanged, but the two run durations are now equal. In scenario 3, $\tau_f = \tau_b = \tau_f^m$, i.e., both run durations equal the measured forward run duration. With $D_r = 0.05\ s^{-1}$ and swimming speed v = 90 µm s^{-1}, we obtain D_1 = 993 µm^2s^{-1}, D_2 = 535 µm^2s^{-1}, and D_3 = 834 µm^2s^{-1}.

The asymmetric durations of forward and backward runs thus increase the diffusion coefficient compared to a symmetric scenario.

Supplementary Note 2

Rheology of mucus and mucin

Many polymer solutions exhibit shear thinning, that is, a decreased viscosity at high shear rates. The shear rate $\dot{\gamma}$ around the rotating flagellum during swimming is $\dot{\gamma} \sim \omega D/d$ where $\omega = 2\pi f$, with f the rotation frequency, D the diameter of the flagellar helix, and d the diameter of the flagellar filament(3). With $D \approx 0.5$ µm, $d \approx 50$ nm, and $f \approx 10^3$ Hz(6), we obtain $\dot{\gamma} \approx 6 \times 10^4$ Hz. While we are not aware of viscosity data for mucus or mucins that cover this high shear regime, extrapolating existing data for mucus(7) or 1.5% porcine gastric mucin(8) is consistent with the expectation that their viscosity approaches the solvent viscosity at this high shear rate. Like PVP K90, mucins have typical radii of gyration similar to or larger than the 50 nm diameter of the flagellum, suggesting that they may also exhibit the nano-scale extreme shear thinning observed by Martinez for PVP K90 (3). The remarkable implication of the work of Martinez et al.(3) is that bacterial motility in strongly shear-thinning solutions may depend only on the macroscopic viscosities of solution and solvent and not on detailed rheological properties, so that the same behaviors are expected for aqueous, strongly shear-thinning solutions of the same macroscopic viscosity. The swimming speed we observe in 1.2% mucin solutions corresponds to that observed in PVP solutions with a macroscopic viscosity of approximately 3 cP.

A number of studies have determined mucin viscosities from the measured diffusion of tracer particles with diameters in the range of 100 nm to 1 µm, similar to the size of a bacterial cell body. Thus, we expect that the resulting values may reflect the macroscopic viscosity experienced by the cell body during locomotion. The reported viscosities range from 1-2 cP for 1.5% human gastric mucin(9) to 3.5 cP at 1.2%(10), 9 cP at 1.5%(11), and 4 cP at 1.2%(12) for porcine gastric mucin, and 4 cP at 1.2% for porcine duodenal mucin MUC2(12). This concentration is well below their entanglement threshold of approximately 2-3% beyond which elastic effects become apparent(12). Measurements for more dilute human MUC5B solutions, however, find viscosities of approximately 4 cP at 0.02% and 8 cP at 0.16%(13). Assuming a uniform power law scaling of viscosity with concentration, these values would imply a viscosity of approximately 15 cP at 1.2%, substantially higher than the estimates for other mucins. In addition to intrinsic differences between mucin species, preparation and handling may also contribute to variabilities.

References

1. Lauga E, DiLuzio WR, Whitesides GM, Stone Ha. 2006. Swimming in Circles: Motion of Bacteria near Solid Boundaries. Biophys J 90:400-412.
2. Schneider WR, Doetsch RN. 1974. Effect of Viscosity on Bacterial Motility. J Bacteriol 117:696-701.
3. Martinez Va, Schwarz-Linek J, Reufer M, Wilson LG, Morozov AN, Poon WCK. 2014. Flagellated bacterial motility in polymer solutions. Proc Natl Acad Sci U S A 111:17771-17776.
4. Tewes M, Peuker UA. 2016. Polymerization in Sprays: Atomization and Product Design of Reactive Polymer Solutions. *In* Fritsching U (ed), Process-Spray.
5. Taktikos J, Stark H, Zaburdaev V. 2013. How the Motility Pattern of Bacteria Affects Their Dispersal and Chemotaxis. PLoS One 8:e81936-16.
6. Magariyama Y, Sugiyama S, Muramoto K, Kawagishi I, Imae Y, Kudo S. 1995. Simultaneous measurement of bacterial flagellar rotation rate and swimming speed. Biophysj 69:2154-2162.
7. Lai SK, Wang Y-Y, Wirtz D, Hanes J. 2009. Micro- and macrorheology of mucus. Advanced drug delivery reviews 61:86-100.
8. Celli JP, Turner BS, Afdhal NH, Ewoldt RH, McKinley GH, Rama Bansil a, Erramilli S. 2007. Rheology of Gastric Mucin Exhibits a pH-Dependent Sol−Gel Transition. Biomacromolecules 8:1580-1586.
9. Su C, Padra M, Constantino MA, Sharba S, Thorell A, Lindén SK, Bansil R. 2018. Influence of the viscosity of healthy and diseased human mucins on the motility of Helicobacter pylori. Sci Rep 8:9710.
10. Celli J, Gregor B, Turner B, Afdhal NH, Bansil R, Erramilli S. 2005. Viscoelastic Properties and Dynamics of Porcine Gastric Mucin. Biomacromolecules 6:1329-1333.
11. Martínez LE, Hardcastle JM, Wang J, Pincus Z, Tsang J, Hoover TR, Bansil R, Salama NR. 2016. Helicobacter pylori strains vary cell shape and flagellum number to maintain robust motility in viscous environments. Mol Microbiol 99:88-110.
12. Georgiades P, Pudney PDA, Thornton DJ, Waigh TA. 2014. Particle tracking microrheology of purified gastrointestinal mucins. Biopolymers 101:366-77.
13. Davies HS, Pudney PDA, Georgiades P, Waigh TA, Hodson NW, Ridley CE, Blanch EW, Thornton DJ. 2014. Reorganisation of the Salivary Mucin Network by Dietary Components: Insights from Green Tea Polyphenols. PLoS One 9:e108372.
